# Emotional social screening tool for school readiness (E3SR-R): Adaptation into Afrikaans

**DOI:** 10.4102/ajopa.v7i0.174

**Published:** 2025-05-19

**Authors:** Erica Munnik, Nuraan Adams, Mario R. Smith

**Affiliations:** 1 Department of Psychology, Faculty of Community and Health Sciences, University of the Western Cape, Cape Town, South Africa

**Keywords:** adaptation, conceptual construct validity, content validity, translation, linguistic equivalence, E3SR-R

## Abstract

The Emotional Social Screening Tool for School Readiness – Revised (E3SR-R) is a contextually sensitive and psychometrically sound measure developed to screen emotional-social competence in preschool learners in South Africa, a multilingual country. The original measure was constructed in English. This article reports on the translation of the E3SR-R into Afrikaans. A three-phase design was adopted. Phase 1: Independent reviewers evaluated the E3SR-R for conceptual validity. The Conceptual Construct Validity Appraisal Checklist was used to assess whether the E3SR-R was theoretically sound prior to adaptation. Phase 2 entailed translation of the E3SR-R. Reviewers used the Quality of Translation and Linguistic Equivalence Checklist to assess compliance with International Test Commission (ITC) guidelines. Phase 3 established content validity of the translation using a Delphi panel of 9 experts. The panel concluded within one round. Ethics clearance was granted by the University of the Western Cape. All applicable ethics principles were upheld. In Phase 1, a high level of inter-rater agreement confirmed that the E3SR had conceptual construct validity that supported adaptation. Phase 2 produced an Afrikaans translation. Raters had a high level of agreement that the adaptation complied with ITC guidelines. The Delphi panel concluded that the Afrikaans version demonstrated content validity. The Afrikaans translation of the E3SR-R was linguistically equivalent.

**Contribution:**

The study employed a rigorous methodology that underscored the importance of establishing conceptual construct validity, evaluating the translation process and establishing content validity in translation studies. Access to screening tools for emotional-social competence in pre-schoolers was expanded.

## Introduction

Ethical practice in testing demands the fair use of psychological assessments across cultures and languages (Hernández et al., 2020). Therefore, the development and adaptation of measures to enhance fair, ethical assessment practice is of vital importance and needs careful consideration (International Test Commission [ITC], 2017). Foxcroft and De Kock (2023) concluded that the development of new, contextually appropriate assessment measures is usually preferred over adaptation. However, in practice, the cost and time investment required for the development of new measures is neither attainable nor sustainable (Laher & Cockcroft, 2014). Thus, there is a growing recommendation for empirically supported, rigorous and methodologically coherent processes for the adaptation of well-designed measures. Laher (2024) recommends selecting contextually sensitive, existing local measures for adaptation in preference to ones developed in other countries.

In South Africa, attempts to reduce bias in assessment practices remain a focus in research (Foxcroft & De Kock, 2023). The vestiges of discriminatory apartheid practices and separate development continue to manifest along racialised, gendered and socio-economic patterns. South Africa is a multilingual country with diverse cultures and religions; this poses significant challenges to the ethical use of measures (Smith et al., 2022). There are 12 official languages (including sign language), which complicates assessment practices (Brenzinger, 2017). The home language of test takers often differs from the language used in their school setting or the communities in which they reside (Reddi, 2024). The implication for test adaptation is that idiomatic expressions included in an instrument, though local, may well be unfamiliar to the target group and may introduce linguistic bias that intersects with models of language, proficiency and multilingualism.

Language is an artefact of culture, and thus cultural bias must be addressed alongside linguistic bias (Van der Merwe et al., 2022). In the South African context, cultural bias can occur when the diverse backgrounds of test users are not considered and the ethnic or cultural context of the test developer or test adapting individual is favoured (Daouk-Öyry & Zeinoun, 2017). Thus, adaptation must be conceptualised as including both translation and establishing linguistic equivalence as complementary processes (Smith et al., 2022). The process of establishing linguistic equivalence must be rigorous and intentional (Daniels, 2020). These processes must be aligned with the ITC guidelines and supported with empirical processes and evidence (Munnik & Smith, 2019). Efforts to adapt, refine and validate local and international measures for use in the South African context remain a focus for ongoing research (Laher, 2024).

### Process of adaptation

Test adaptation is usually described as a multi-layered process that comprises adaptation, translation and validation (Hernández et al., 2020). Adaptation focuses on the adjustment of the content of the measure to enhance cultural suitability and accuracy (Iliescu et al., 2024). The translation process entails converting the test content from an original or source language to a target language (Özturk et al., 2015). Translation usually follows a sequence of carefully designed steps, including forward- and back-translation to achieve linguistic and semantic equivalence (Hambleton & Zenisky, 2011). Linguistic and semantic equivalence entails the processes used to ensure that the translated or adapted version measures the same constructs and retains the same meaning as the original version (Iliescu et al., 2024). Equivalence is considered a critical aspect of the translation and adaptation processes, as it confirms that the modified test remains psychometrically sound and produces dependable results across the different linguistic forms of the assessment measure. Validation of the adapted or translated version establishes that the measured constructs and content are still illustrative of the domains of the measure (Behr, 2017). This article reports on the translation of the Emotional Social Screening Tool for School Readiness (E3SR), a screening tool for social-emotional competence as a domain of school readiness.

### Contextualising schooling and readiness for schooling in the South African context

In South Africa, according to the *Basic Education Laws Amendment Act* (Republic of South Africa, 2024), children are required to enrol in mainstream or formal education (Grade 1) in the year they turn 7 years old. There is no mandatory assessment of school readiness, as chronological age is used as an indicator of developmental maturity; thus, 7-year-old children are expected to possess the skills and competencies required to engage successfully in learning and formal instruction. The Act further makes attendance of the reception year (Grade R) mandatory; Grade R precedes Grade 1 (Republic of South Africa, 2024). This reception year is intended to address inequality and vulnerabilities stemming from adverse socio-cultural, economic and less-stimulating early academic environments, among others (Feza, 2015).

In South Africa, Grade R has a play-based learning curriculum designed to strengthen basic language, arithmetic and life skills as a bridge to the formal instruction that begins in Grade 1 (Feza, 2015). Thus, it is expected of young learners to be physically, cognitively, emotionally and socially ready to start formal schooling after they complete Grade R successfully. The Department of Basic Education (DBE) (Department of Basic Education [DBE], 2016) prescribes a formal assessment of Grade R learners’ cognitive, emotional and social skills within the curriculum assessment policy (DBE, 2016). However, this assessment places greater emphasis on the cognitive domains of the learner’s readiness (Mtati & Munnik, 2023); there is thus a need for a complementary assessment of emotional and social skills as domains of school readiness (Munnik et al., 2021).

### Emotional social screening tool for school readiness – Revised

The E3SR-R is a paper-and-pen, English, Likert-type screening tool used by professionals (e.g., educators) to evaluate emotional and social competence as a domain of school readiness in preschool children (Munnik, 2018). The E3SR-R can be used in a summative or formative manner to assess strengths and areas of growth in social-emotional competence in preschool learners that can complement cognitive measures and assist with decisions about entry into formal schooling (Grade 1). During the past 5 years, the original E3SR was revised from 56 items to the current 36-item E3SR-R (Koopman et al., 2024). The E3SR-R was found to be a reliable and valid instrument for use in the South African context, as evidenced by good psychometric properties and model fit indices (Munnik et al., 2021). The need to broaden the E3SR-R’s use in South Africa’s multilingual educational settings was identified after its revision in 2021 (Koopman et al., 2024). The translation of the E3SR-R would respond to the multilingual South African context (Reddi, 2024). This article reports on the translation process of the E3SR-R into Afrikaans, the second-most-spoken language in the Western Cape region where the researchers are located.

### Aim of the study

This article reports on the adaptation of the E3SR-R (English version) through translation into Afrikaans.

## Methods

The study was conducted in three phases, namely construct validation, translation and content validation. Each phase had its methodological elements.

### Phase 1: Conceptual construct validation

The E3SR-R was selected as a locally developed instrument with established psychometric properties reported (Munnik et al., 2021). The first step of the adaptation entailed a qualitative investigation of the conceptual construct validity of the E3SR-R. The ITC (2017) recommended that the conceptual validity of instruments must be examined in addition to data-driven, reductionist metrics of construct validity. Conceptual construct validity is viewed as the cogent underpinning of instrument development (Villarino, 2024).

This non-reactive phase entailed an evaluation of the process followed in conceptualising the E3SR. The objective of this phase was to ascertain whether construct validity was achieved in the development of the E3SR, before proceeding with translation. Two independent fieldworkers were recruited to rate the conceptual process using the Conceptual Construct Validity Appraisal Checklist (CCVAC). The CCVAC was used to evaluate the rigour of the process of conceptually defining the construct for operationalisation or measurement.

The CCVAC, developed by Smith and Munnik (2021), operationalises Rossiter’s (2016) construct definition, object classification, attribute classification, rater identification, scale formation, and enumeration and reporting (C-OAR-SE) theory of conceptual validity. The CCVAC has three sections, assessing theoretical definitions, operational definitions and technical aspects such as scoring (Smith & Munnik, 2023). The developer of the E3SR completed the CCVAC template in which a detailed account was created of information on the process of defining the construct being measured by the E3SR, including how theoretical and operational definitions were derived. This template structure facilitates ease of evaluation when using the CCVAC and ensures that lack of familiarity with the underlying theory does not adversely influence the evaluation process. Two research psychologists with training in research methods and test construction completed the CCVAC rating independently. The ratings were scored using the scoring system of the CCVAC. This produced subsection scores, section scores and a global score. The interpretation matrix for the CCVAC was used to interpret all scores and derive a sense of the extent to which conceptual validity was achieved. All resultant scores were subjected to a correlation to establish inter-rater reliability.

### Phase 2: Translation and linguistic equivalence

In this phase, the E3SR-R was translated into Afrikaans. The translation followed the first five steps of adaptation proposed by Sousa and Rojjanasrirat (2011). Step 1 entailed the translation of the E3SR-R into Afrikaans. All the translators had to satisfy the following criteria: be fluent in Afrikaans, hold a qualification in editing and language studies and have experience in translation. Two translators were used, and the resultant Afrikaans’ drafts (TL-1 and TL-2) of the E3SR-R were captured onto a columned template. The first two columns contained the content of TL-1 and TL-2, respectively.

Step 2 entailed comparison of the two translated (Afrikaans) versions (TL-1 and TL-2), using the abovementioned template. Column 3 made provision for recording the level of agreement between the two translations, and Column 4 captured the preferred translation. Two reviewers were used in this process. Firstly, Reviewer A selected the most suitable translation for each of the items from the two translations. The reviewer identified identically translated items first and transferred them to the draft Afrikaans translation (TL-3). If translations were equivalent, but different phrasings and expressions were noted, the reviewer selected the most appropriate version to transfer to TL-3. This resulted in a draft version of the E3SR-R in the target language. Secondly, Reviewer B evaluated the recommendations of the first reviewer. Any disagreements were discussed until a consensus was reached. This resulted in a prefinal draft translation in the target language (TL-3).

In Step 3, the draft translation (TL-3) was translated back into English by three different translators producing three back-translations (BTL-1, BTL-2 and BTL-3). Similarly, to the forward-translation, they were provided with a template containing the Afrikaans segments from TL-3 and a space for the translation output (i.e., segments in English). This step resulted in three back-translated versions. In Step 4, the three back-translated (English) versions were compared by the same Reviewer (A) used in Step 2 to ensure optimal familiarity with the original version. The template was expanded to six columns to capture the comparison of the three back-translated versions. The first column contained the segments from the original English source-language version of the E3SR-R. The second to fourth columns contained the segments from BTL-1, BTL-2 and BTL-3. The fifth column provided space for recording Reviewer A’s decisions and the sixth column recorded the recommendations of Reviewer B. Table 1 provides a summary of the four-code key that was used by the reviewers to record the outcomes.

**TABLE 1:** Coded key used to record the outcome of the reviewer comparisons.

Code	Description	Conclusion	Outcome action
1	Meaning was captured clearly and consistently.	Translations are equivalent.	No action required.
2	Meaning was captured, but idiomatic expression differs.	Different phrasings and colloquial expressions are possible, but translations are equivalent.	Determine most appropriate version. Assess source document for possible differences in interpretations.
3	Meaning of constructs differs.	The source and target versions may differ significantly.	Revisit both documents and assess for possible revision.
4	The translations offered a more refined linguistic phrasing than the original.	Clearer meaning or expression derived from comparisons.	Refine source document or target document.

In Step 5, the quality of translation was assessed and the process for establishing linguistic equivalence was evaluated. This process was guided by the Quality of Translation and Linguistic Equivalence Checklist (QTLC), developed by Smith et al. (2022). This assessment was conducted in two steps. Initially, the developer of the E3SR-R completed the QTLC template by recording information about the translators, the process followed in translation, the process for dealing with any discrepancies that arose during the translation process and if verification was sought after resolution of differences. This template provided the data for evaluating the translation process. Two independent researchers rated the translation process using the QTLC. The QTLC consists of two sections that evaluate translation and linguistic equivalence, respectively. Section scores were produced that were interpreted using the QTLC scoring guide. Qualitative descriptions are derived for each section (i.e. translation and linguistic equivalence) (Smith et al., 2022). After completion, the raters submitted their reviews to the research team. The scores were compared and discussed, and any differences were resolved among the research team. Thereafter, inter-rater reliability was computed by means of a Kappa coefficient.

### Phase 3: Content validation

This phase established content validity for the newly translated Afrikaans version of the E3SR-R. A panel of nine experts in developmental psychology and/or test construction was recruited to participate in a Delphi process. The Delphi technique is an iterative process in which the Afrikaans E3SR-R was presented as a stimulus document on the Google Forms platform. The first section of the stimulus document addressed the purpose of the Delphi study and explained the structure of the E3SR-R. The second section presented the definition of each of the domains and subdomains, accompanied by their attributes (in English) for ease of reference. The E3SR-R items under each subdomain were presented in Afrikaans and English. The stimulus document included prompt questions such as: ‘Please tick each item that appropriately measures and reflects the definitions of this subdomain and attributes’ and ‘Please suggest any revisions in phrasing of the identified items’. The panel was also allowed to provide qualitative comments and make recommendations to ensure a critical engagement with the process.

A trial administration to pilot the stimulus document allowed for revision of the content and structure, including correction of grammatical errors, and the revision of technical aspects such as ensuring that the content was in a readable font. Once the suggested changes were implemented, a second trial run was conducted to ensure that the stimulus document was ready for circulation to the identified panellists. After circulation to the panellists, a 2-week period was allowed for completion. Thereafter, a reminder was sent to the panellists who did not respond. A threshold of 70% was set for consensus among panellists, in keeping with the recommendation of Naisola-Ruiter (2022) who identified a level of consensus above 70% as a strong level of agreement. Responses were tallied and expressed as a percentage of agreement. Thereafter, the consensus agreements were categorised as ‘strong’ (> 70%), ‘fair’ (50% – 70%) or ‘low’ (< 50%) levels of agreement. Items that attained a consensus above 70% were retained unchanged. Items that obtained a low level of agreement were earmarked for replacement, revision or omission.

### Ethical considerations

Ethics approval was granted by the Humanities and Social Science Research Ethics Committee of the University of the Western Cape (HS21/9/2). The three phases of the study conformed to all ethics guidelines including, but not limited to, anonymity, confidentiality, informed consent, voluntary participation and the right to withdraw from the research at any time if necessary. All data collected were securely stored, hard copy documents were secured in a locked cabinet and electronic files were stored in a password-protected folder.

## Results

### Phase 1: Conceptual construct validity for the Emotional Social Screening Tool for School Readiness

#### Global score

The scores for Rater 1 suggested that the E3SR-R attained a medium level of conceptual construct validity, whereas the scores for Rater 2 suggested a high level of conceptual construct validity. These qualitative descriptions were derived from the scores attained across the three sections of the E3SR-R. The difference between the two ratings became clear following an exploration of the section scores. Thus, the scores indicate that the E3SR-R achieved a moderate-to-high level of conceptual construct validity.

#### Section 1: Theoretical definition

Both raters awarded the maximum composite score (*n* = 3) for their evaluation of the theoretical definition provided in the E3SR-R. The CCVAC composite score suggested that the E3SR-R included sound conceptual definitions of the construct being measured.

#### Section 2: Operational classification

The section scores for the two raters differed by three points, which resulted in different composite scores being derived. Rater 1 awarded the maximum score for this section (*n* = 13), with a section score of two; this suggests that the operational definitions were founded on partially correct classification. The subsection scores for Rater 2 (*n* = 16), with a section score of three, suggest that the operational definitions were founded on sound and correct classification of the construct and its attributes. Upon closer examination, it became evident that the discrepancy between the ratings was attributable to one item. A follow-up engagement with the raters revealed that the second rater misunderstood the item. For the purposes of the analysis, it was concluded that the development of operational definitions in the E3SR-R was founded on sound and correct classification of the construct.

#### Section 3: Technical aspects

The section scores for the two raters differed by 2 points, which resulted in different composite scores being derived. Rater 1 awarded the maximum score for this section (*n* = 13); this articulated into the maximum composite score (*n* = 3), suggesting that the E3SR-R included sound technical scalar decisions. The score for Rater 2 (*n* = 11) articulated into a lower composite score (*n* = 2), suggesting that the scoring of the E3SR was based on partially sound technical scalar decisions. Upon closer examination, it became evident that the discrepancy between the ratings was attributable to one item. A follow-up engagement with the raters revealed that the scores of the second rater followed on from the misunderstood item that was scored lower in Section 2. Thus, the scoring was consistent, albeit incorrectly scored lower. For the purposes of the analysis, it was concluded that the development of scoring of the E3SR-R was founded on sound technical scalar decisions.

The exploration of the section scores revealed that the scoring was consistent. The observed differences in ratings were explained, and it was concluded that the global scores suggested that the E3SR-R attained a sufficient level of conceptual construct validity. Both scores recommend that the E3SR-R may be subjected to psychometric testing. The lower global score derived from Rater 2 suggested that caution must be applied when proceeding with psychometric testing. For the purposes of this study, the results were interpreted to mean that the E3SR-R had a level of conceptual construct validity that would support the establishment of psychometric properties.

#### Inter-rater reliability

The Kappa statistic (0.554) tested significantly at a 0.01 alpha level (*p* = 0.002), indicating a significant, moderate agreement between the raters.

### Phase 2: Translation of the Emotional Social Screening Tool for School Readiness into Afrikaans

#### Step 1: Translation from the source to the target language

Two translators translated the E3SR-R from the source language (English) into the target language (Afrikaans). Table 2 summarises the demographics of the two translators.

**TABLE 2:** Demographics of the translators.

Translator	1	2
Profession	Clinical psychologist	Research psychologist
Area of expertise	Clinical practice, language editing and translation	Instrument development and research
Qualification in editing	Yes	Yes
Language studies	Yes	No
Psychology	Yes	Yes
Development studies	Yes	Yes
Years of experience in Translation	45	40

The translators were both established researchers with prior knowledge and relevant experience in translation studies. They were fluent in Afrikaans and well acquainted with developmental psychology.

#### Step 2: Comparison of the translated versions (TL-1 and TL-2)

Table 3 portrays a summary of the results of the forward-translation process. For ease of reporting, the demographic section and screening items of the E3SR-R are presented separately. Table 3 identifies the agreement between translations and decision-making about translated items.

**TABLE 3:** Results of the review process.

Concordance in translation	Section		Recommendation		Decision
	Demographic	E3SR	Reviewer 1	Reviewer 2	
Items translated identically	20	30	Adopt into TL-3 draft	Endorsed	Adopted into TL-3 draft
Items translated equivalently but with different phrasing	21	21	Identified most appropriate versions	Endorsed	Recommended version adopted into TL-3 draft

**Total**	**41**	**51**	-	-	-

E3SR, Emotional Social Screening Tool for School Readiness.

A total of 50 items and phrases were translated identically and adopted into the third translation draft (TL-3). After comparison of all the items and phrases that were translated equivalently, but with different phrasings, 42 preferred versions of the translations were adopted into the TL-3. The TL-3 represented the prefinal draft of the Afrikaans translation and was subjected to the back-translation process.

#### Step 3: Back-translation of draft translation (BTL-1, BTL-2 and BTL-3)

Three independent translators managed the back-translation, resulting in three draft versions (BTL-1, BTL-2 and BTL-3). Table 4 summarises the demographics of the three translators.

**TABLE 4:** Demographics of the back-translators.

Profession and qualification	Translator		
	1	2	3
Profession	Research psychologist	Clinical psychologist	Linguist
Qualification in area of expertise	Research and capacity building	Clinical practice, Attention Deficit Hyperactivity Disorder, Language editing	Language education and communication studies, Translation
Editing	Yes	No	Yes
Language studies	Yes	No	Yes
Psychology	Yes	Yes	No
Development studies	Yes	Yes	Yes
Years of experience in Translation	3	4	30

ADHD, attention deficit hyperactivity disorder.

#### Step 4: Comparison of the back-translated versions

The results of the review process for the back-translation are displayed in Table 5.

**TABLE 5:** Back-translation review process (*N* = 92).

Code	Qualitative description	Interpretation	Number of items or phrases		Decision
			Demographic (*n* = 41)	Screening (*n* = 51)	
1	Meaning was captured clearly and consistently.	Translations are equivalent.	20	30	Retain items.
2	Meaning was captured, but idiomatic expression differs.	Translations are equivalent, but different phrasings and colloquial expressions were used.	16	13	Retained the items in the Afrikaans source.
3	Meaning of constructs differs.	Afrikaans source document may be different from English source.	4	3	Afrikaans source document was revised.
4	The translations offered a more refined linguistic phrasing than the original.	Clearer meaning or expression derived from comparisons.	1	5	Item in English source document was revised to adopt phrasing in BTL-1.

From Table 5, it becomes evident that 20 items in the *demographic* section were back-translated with consistency between the translators, suggesting that the meaning was clearly captured between the translations. Thus, these items were retained in the final Afrikaans version. The BTL included 16 items where idiomatic expressions differed, but the meaning was equivalent. Thus, different versions were possible without losing or distorting the meaning of the items in the prefinal Afrikaans version. The 16 items in the prefinal Afrikaans version were thus retained and adopted in the final Afrikaans version. Thus, a total of 36 items in the demographic section of the prefinal Afrikaans version were adopted in the final Afrikaans version.

Four items were translated differently in the three BTL, suggesting that these items in the prefinal Afrikaans version (source) differed from the corresponding items in the original English source. For example, ‘Personal particulars of learner’ (English source) was back-translated as ‘Personal information of learner’ (BTL-1), ‘Personal details of learner’ (BTL-2) and ‘Learner’s personal information’ (BTL-3), respectively. The lack of consistency in the BTL prompted the reviewers to consider the phrasing in the source document. The reviewers concluded that the source document must be revised. As a result, the phrasing ‘Personal information of learner’ was adopted as a revision in the E3SR-R English source version, as it is a more common colloquial phrase. Fundamentally, the meanings in context were maintained. The four items in the Afrikaans version were revised to achieve greater clarity of meaning. The translation of one item in one of the BTL-1 offered a clearer meaning or expression than the English source. This phrasing was refined in the English source for clarity.

From Table 5, it becomes evident that 30 items in the *screening section* were back-translated with consistency between the translators, indicating that the translations clearly captured the meaning of the item in the source documents. Thus, these items were adopted in the final Afrikaans version. The BTL included 13 items translated with different idiomatic expressions. Despite the differences in expression, the meanings of the items were equivalent, and no meaning was distorted in the BTL. The 13 items in the prefinal Afrikaans version were thus adopted in the final Afrikaans version. Thus, a total of 46 items in the screening section of the prefinal Afrikaans version were adopted in the final Afrikaans version.

Three items were translated differently in the three BTL. These items in the prefinal Afrikaans version differed from the corresponding items in the original English source. For example, reviewers noted that item 58 was misinterpreted by all translators. The item read: *‘Aanvaar teregwysing/dissipline*’, which was interpreted by translators as ‘Accepts reprimand/discipline’ (BTL-1), ‘Accepts discipline’ (BTL-2) and ‘Accepts admonishing/discipline’ (BTL-3), respectively. Both reviewers noted these differences and concluded that ‘*teregwysing*’ [reprimand] should be replaced with ‘*korreksie*’ [correction] in the Afrikaans draft. The three items in the Afrikaans version (Items/Phrases 59, 65 and 68) were revised to clarify the meaning and make them more equivalent to the English source. Five items in BTL-1 (Items/Phrases 44, 45, 56, 58 and 62) were expressed more clearly than the corresponding item in the English source. The phrasing in BTL-1 for those items was adopted in the English source for clarity.

#### Step 5: Assessing the quality of the translation and equivalence process

Two raters completed the QTLC independently. Rater 1 (R1) was a research psychologist with proficiency in the field of statistical practice that included 5 years of experience in translation and equivalence studies and in psychometric test development. Rater 2 (R2) was a research psychologist with expertise in the field of capacity development and transferable skills training in research methodology. R2 did not have any experience in translation but possessed 3 years of experience in equivalence studies.

The raters awarded identical scores on the subsections of the *Translation process* as measured by the QTLC. The composite score of 24 indicates that a high level of compliance was achieved with the ITC guidelines in the translation processes. Thus, the establishment of equivalence could be commenced. The composite scores for *linguistic equivalence* differed by 4 points between the two raters. The raters awarded identical scores (*n* = 15) in the subsection that evaluates the process followed to compare the source and target translations. The raters awarded different scores on two items in subsection 2 (comparison between target and back-translation) and two items in subsection 3 (comparison between source and back-translation). It appeared that these items had differential functioning, resulting in the difference in the scores awarded. This limitation was offset by following up with the raters to confirm their scoring after clarifying the item. Rater 1 adjusted her scoring, which resulted in both subsections having identical scores of 12. The revised composite score (*n* = 43) for linguistic equivalence indicated a high level of compliance with ITC guidelines related to the processes followed when establishing linguistic equivalence. Thus, the establishment of reliability was recommended.

The ratings were tabularised and inter-rater reliability was calculated. The Kappa statistic (0.78) tested significantly at an alpha level below 0.01 (*p* = 0.000). The statistic exceeded the minimum threshold score (0.61) for acceptable inter-rater reliability coefficients, as recommended in the seminal article by Hallgren (2012). The finding suggests that there is a substantial agreement between raters on the quality of the translation process and equivalence.

### Phase 3: Content validity

The Delphi panel was comprised of nine panellists (seven female and two male panellists) with expertise in the areas of psychology and education. Most of the panellists had more than 6 years of experience in their respective vocations. All panellists were fluent in Afrikaans, and the majority were mother tongue speakers. Table 6 provides an overview of their demographics.

**TABLE 6:** Demographics of panellists.

ID	Area of expertise	Job title or vocation	Experience in years
1	Child development	Lecturer	16–20
2	Educational assessments	Psychometrist	6–10
3	Therapy	Psychologist	6–10
4	Child development Therapy	Psychologist	1–5
5	TeachingTherapy	Psychologist Academic	6–10
6	Therapy	Psychologist	> 21
7	Therapy	Psychologist	> 21
8	Therapy	Psychologist	1–5
9	Teaching and Administration Child development	Principal	> 21

ID, identification.

The results of the first round reflected that the panellists’ responses exceeded the 70% threshold level of consensus for all items in the Social Competence domain, comprising Social Skills and Communication. Similarly, consensus was reached on all items in the Emotional Competence domain, comprising Emotional Maturity, Emotional Management and Sense of Self.

In the Readiness to Learn subdomain, only item 3 attained a score below 70%. Six of the nine panellists (66.7%) felt that item 3, ‘Can attend and focus on a task’, should not be included in the Readiness to Learn subdomain. They suggested a revision of the item. One panellist advised that readiness to learn also forms part of children’s interest or curiosity to learn. Another panellist advised that the researcher should add more context to the item and suggested the following revision: ‘Pay attention and can focus on a task even if he/she does not necessarily feel like the activity’. The proposed revision would have changed the meaning of the item, which was not in line with the subdomain’s definition and attributes. For example, the original item focused on the actions that the child would be capable of, whereas the proposed revision introduced motivation. Motivation was related to Emotional Management but did not speak to this subdomain and its attributes. The research team decided to retain the item in its original format.

The Delphi study was concluded in one round because of the high level of agreement among the panellists. Thus, the findings and the high level of agreement suggested that the translation had face and content validity.

## Discussion

### Phase 1: Conceptual construct validity

The E3SR-R was found to have conceptual construct validity. Notwithstanding the discrepancy noted between the raters in two sections, construct validity for the E3SR was confirmed. The clarification sought from the raters approximated member checking, which was beneficial in three ways. Firstly, the ‘rater checking’ increased the rigour of the study. Secondly, it enhanced the validity of the findings. Thirdly, it enabled the researchers to make informed decisions that were verified by the raters rather than being subject to researcher bias. Thus, the use of the CCVAC provided empirical data for the summative evaluation of conceptual construct validity. The subsection and section scores provided a more refined level of analysis that guided decision-making, as recommended by Smith and Munnik (2023).

The Kappa coefficient provided an indication of inter-rater reliability. The Kappa coefficient exceeded the threshold levels recommended by Hallgren (2012) and indicated a high level of inter-rater reliability. The findings of this phase provided a strong evidence base for proceeding with the translation of the E3SR-R into Afrikaans (Phase 2).

The inclusion of this first phase is important in two ways. Firstly, it underscores the importance of evaluating conceptual construct validity before proceeding with the traditional data reduction techniques typically used to assess construct validity. Villarino (2024) cautioned that factor analytic techniques are seen as the gold standard when evaluating construct validity, with the focus on model fit. However, if the measure lacks conceptual construct validity, the resultant model fit indices lose their value and robustness. The importance of clarifying conceptual construct validity is illustrated by the methods and instrumentation used in this phase. Secondly, the instrumentation used in the study provides empirical support for what previously was treated as theoretical and therefore assumed to be less valuable than statistics and metrics from an ‘evidence’ point of view. Smith and Munnik (2023) developed the CCVAC, which offered an operationalisation for measuring construct validity. The CCVAC, an empirically tested and theoretically grounded instrument, provided a quantifiable measure of conceptual validity. The process of this phase confirmed that while the instrument was underpinned by Rossiter’s C-OAR-SE theory, prior knowledge of the theory per se was not required for it to be used meaningfully and effectively. These process observations confirm the claims of Smith and Munnik (2023).

### Phase 2: Translation

The explicit and detailed reporting of the translation process was an important methodological decision in the execution and reporting of the study phase. The steps and decision-making that directed the translation process were clearly articulated, which enhanced the replicability of the study phase. Iliescu et al. (2024) reflected that translation is often reported in an overly simplified manner that does not provide adequate information to enable the evaluation of methodological rigour and coherence. The Phase 2 steps were consistent with the recommendations contained in the ITC guidelines. The translation included both forward and backward translation, consistent with best practice as recommended by Iliescu et al. (2024). The use of three translators in the backward translation was above-threshold practice in comparison with the standard two translators (ITC, 2017). Similarly, highly qualified and experienced translators were used, which resulted in refinement of the source document. Iliescu et al. (2024) argued that good translation processes have the capacity to inform revisions of the original or source versions; however, this is seldom achieved because of the lack of rigour applied to the translation process. Factor analyses are often favoured at the expense of thoroughness in the translation process. Phase 2 attempted to heed this caution in the process of translation through careful planning and explicit decision-making; this in turn made reporting more feasible and reliable.

The explicit reporting on the template for recording the translation process and demonstration of how decision-making could be captured for posterity and evaluation was useful. The template and its adaptability for use in the forward and backward translation process provided a clear way to track the process. In addition, the sequential review process demonstrated a time- and resource-efficient way to use reviewers and provided clear opportunities for recording the recommendations. Furthermore, the code or key used made decisions explicit and guided reviewers and researchers to reach conclusions and make decisions about the resultant translations that in turn enhanced the possibility of achieving linguistic equivalence. The sequential vetting process used by reviewers demonstrated the progression from translation of a phrase or item to adoption into the draft and final Afrikaans versions of the E3SR-R. This constitutes process data that are often overlooked in the reporting and perhaps even in the conceptualisation of adaptation studies using translation.

In Phase 2, linguistic equivalence was established. The results from the QTLC suggested that the process followed in the translation of the E3SR-R evidenced a systematic process. The raters had a high level of agreement as evidenced by the calculated inter-rater reliability. The QTLC scores suggested a high level of alignment with the ITC guidelines for adaptation through translation. The use of the QTLC provided a structured, empirical assessment of the test adaptation practices. The instrument assisted with accurate and transparent reporting of the decisions made and the steps operationalised in the translation of the E3SR-R into Afrikaans, as recommended by Smith et al. (2021).

The checklist constituted an operationalisation of the ITC guidelines for good practice in translation and establishing linguistic equivalence. This checklist creates a means for empirically evaluating the translation process from a theory-driven perspective that produces quantifiable outcomes. The checklist contributes to making the methodology underpinning translation unambiguous. This improves upon the implicit assumptions offered in the reporting of adaptation studies, thus providing empirical evidence for processes that were historically only described in a qualitative way. The findings of the QTLC also confirmed that linguistic equivalence was achieved. This phase made clear recommendations supported by empirical data that the process for establishing psychometric properties of the translated E3SR-R could proceed.

### Phase 3: Validation

This phase presented a panel of nine experts with the original and translated versions for validation. The E3SR-R Afrikaans version was found to have face and content validity. Delphi techniques are typically concluded in a minimum of two to three rounds (Naisola-Ruiter, 2022). In this instance, the high level of consensus established among the panellists resulted in the conclusion of the Delphi method in a single round; this is in part attributable to the decision to engage with conceptual validity in Phase 1 and the systematic and rigorous translation process in Phase 2, as precursors to the establishment of content validity. The Delphi proved invaluable to leverage the contribution of the panel of experts.

## Conclusion

The multiphased nature of the design proved beneficial as it allowed each phase to adopt specific methodologies without forcing an ill-fitting overarching strategy. The triangular nature of the adaptation process strengthened the overall outcomes. It is important to note that the overarching design was not conceptualised as a mixed-methods design. Phase 1 emphasised the importance of establishing conceptual validity as a precursor to data reduction techniques such as factor analysis, which are still largely used as the preferred method to establish construct validity in measurement research. The use of the CCVAC and the QTLC proved valuable and added to the rigour of the adaptation (translation) process. Both instruments provided alternative ways of engaging with different aspects of the adaptation process without the need to understand and engage with the theoretical underpinning of the instruments.

The translation was concluded using a detailed and stringent process with explicit operational steps that enhanced the level of methodological rigour, thus underscoring the importance of a rigorous and thorough process in translation and equivalence studies. The study broadened methodological thinking in test adaptation through the use of instrumentation for aspects that, historically, were treated qualitatively and/or given token attention in the methodology of adaptation studies. The adaptation of the E3SR-R through translation into Afrikaans extends the culture-fairness of screening for social-emotional competence in young learners in South Africa.

### Limitations

The CCVAC and QTLC appeared to have items that functioned differently, as evidenced by the scores allocated by the raters and the clarification during the rater checking. The impact of this differential item functioning was limited in this study through rater checking. Refinement of the identified items on instruments is thus essential, as misinterpretation of items needs to be restricted. Feedback was provided to the authors and developers of the instruments.

### Recommendation

This study should be replicated to facilitate the translation of the E3SR-R into other official languages, such as isiXhosa. IsiXhosa is the most spoken and understood language among South Africans in the Western Cape. To avoid linguistic constraints for isiXhosa-speaking test-takers whose mother tongue is neither English nor Afrikaans, the E3SR-R should be made available in isiXhosa, using the methodological process followed within this study.
